# Social, cultural and community engagement and mental health: cross-disciplinary, co-produced research agenda

**DOI:** 10.1192/bjo.2020.133

**Published:** 2020-12-01

**Authors:** Daisy Fancourt, Kamaldeep Bhui, Helen Chatterjee, Paul Crawford, Geoffrey Crossick, Tia DeNora, Jane South

**Affiliations:** Department of Behavioural Science and Health, University College London, UK; Centre for Psychiatry, Barts & The London School of Medicine and Dentistry, Queen Mary University of London, UK; Research Department of Genetics, Evolution and Environment, Division of Biosciences, University College London, UK; Institute of Mental Health, University of Nottingham, UK; School of Advanced Study, University of London, UK; Department of Sociology, University of Exeter, UK; School of Health & Community Studies, Leeds Beckett University, UK

**Keywords:** Psychosocial interventions, community mental health, research agenda, social, cultural

## Abstract

**Background:**

There is increasing cross-disciplinary research on the relationship between individuals’ social, cultural and community engagement (SCCE) and mental health. SCCE includes engagement in the arts, culture and heritage, libraries and literature, sports and nature activities, volunteering, and community groups. Research has demonstrated the effects of these activities both on the prevention and management of mental illness. However, it remains unclear whether current research is focusing on the research questions that are of most immediate urgency and relevance to policy and practice.

**Aims:**

The current project was funded as part of the UK Research and Innovation cross-disciplinary mental health network programme to develop and co-produce a new cross-disciplinary research agenda on SCCE and mental health.

**Method:**

Established processes and principles for developing health research agendas were followed, with a six-phase design including engagement with over 1000 key stakeholders, consultations, integration of findings and collective prioritisation of key questions.

**Results:**

We identified four core themes: the mode of engagement, process of engagement, impact of engagement and infrastructure required to facilitate engagement. There were many points of agreement across all stakeholder groups on the priority questions within these themes, but also some specific questions of relevance to different sectors.

**Conclusions:**

This agenda is particularly timely given the extreme pressure on mental health services predicted to follow the current COVID-19 pandemic. It is important to identify how resources from other sectors can be mobilised, and what research questions are going to be most important to fund to support SCCE for mental health.

## Community assets and mental health

Over the past decade, there has been a growing focus on the resources (or ‘assets’) that exist within communities and how they can support health and well-being.^1^ Community assets in the broadest sense can include the skills and knowledge of individual community members, local groups and community and voluntary associations; the public-, private- and third-sector resources and facilities within communities; and physical, environmental and economic resources.^1^ Action to surface and mobilise such community resources for health and well-being as well as other purposes is sometimes referred to as an ‘asset-based approach’.^2,3^

One of the areas of asset-based research that has received increasing attention in recent years is the role of community assets in leisure behaviours, including enabling and determining people's social, cultural and community engagement (SCCE).^1^ SCCE can include activities that require active participation (e.g. participating in arts, literature, sports, volunteering, nature activities or community groups) and those that involve a broader type of engagement with community assets (e.g. visiting heritage sites, museums, libraries and spending time outdoors in green spaces). More specific examples of SCCE are shown in Table 1.

**Table 1:** Examples of social, cultural and community engagement (SCCE)

Example types of SCCE	Example activities
Arts	Engaging in groups relating to performing arts (e.g. music/dance/theatre) and groups relating to visual arts and crafts (e.g. textiles/drawing/woodwork/painting/photography/ceramics/sculpture)
Culture and heritage	Going to museums, galleries or exhibitions; the theatre or concerts; the cinema; festivals, fairs or events; stately homes or buildings; historical sites; and landscapes of significance
Libraries and literature	Going to libraries, visiting archives and being a member of book clubs or writing groups
Sports and nature activities	Going to parks or gardens, engagement with allotments or gardening groups, joining nature walks or rambling groups, participation in exercise classes, membership of sports clubs, participating in community sporting activities and attending amateur or professional sporting events
Volunteering	Charitable volunteering, conservation volunteering or school or community volunteering
Community groups	Engaging with education or evening classes; youth services (e.g. Scouts and Guides); political, trade union or environmental groups; resident groups; religious or faith groups; and social groups (e.g. mum and baby groups, cookery groups and other clubs)

There are estimated to be over 1 million assets within communities in the UK that support these types of SCCE: over 40 000 choirs, 11 000 amateur orchestras, 50 000 amateur arts groups, 5000 amateur theatre societies, 3000 dance groups, 2500 museums, 400 historic places, 4000 libraries, 1300 theatres, 50 000 book clubs, 27 000 public parks, 1000 community gardens, 6500 leisure centres, 151 000 sports clubs, 10 000 village halls, 330 000 allotments, 161 000 voluntary associations and 160 000 community groups.^4^ These assets are generally shaped and funded not by the health or social care sector, but by the creative, cultural, community and environmental sectors and by communities themselves. However, they have been shown to act as in-kind resources to health and social care through the impact they can have on mental health. From a prevention perspective, there is a rich research literature on SCCE and hedonic, eudemonic, social well-being and the prevention of mental illness.^5–11^ From a treatment perspective, specific programmes involving SCCE have also been developed for a wide range of mental illnesses, including psychotic disorders, addiction recovery, mood disorders, anxiety disorders and personality disorders.^7,12–15^ Further, we have an increasingly sophisticated understanding of why we find these effects, with a recent review identifying over 600 mechanisms of action.^16^ Some of the best-evidenced mechanisms include psychological processes (such as supporting emotion regulation, building resilience, developing identity and enhancing cognitive resources), biological processes (such as modulating brain activity, altering levels of hormones, increasing physical performance and mitigating biological weathering), social processes (such as increasing social connections, developing supportive relationships and enhancing social cohesion) and behavioural processes (such as increasing behavioural activation, providing motivation, increasing agency and establishing habits).^16^

Alongside this research, there are growing developments in policy and practice relating to SCCE. For example, over the past decade, a number of different countries have been piloting social prescribing schemes that connect patients to non-medical sources of support within the community, such as social, cultural and community activities.^17–19^ These schemes have particularly sought to target individuals with mild-to-moderate mental illness or related social problems such as loneliness as a way of tackling these complex issues and reducing demand on health services.^17–19^ In the UK, these pilots have been developed into a national roll-out, with aims for around 1 million patients a year to be going through the scheme by 2023–2024.^20^ However, despite the high level of activity in this field of research and in policy and practice, to date, much of this effort has been uncoordinated. As such, it remains unclear whether current research is focusing on the research questions that are of most immediate urgency and relevance to policy and practice. Therefore, there is a need to identify and prioritise key research questions that hold the most importance and practical value to different stakeholders.

## Research agendas

Over the past few years, a number of research agendas around mental health have emerged, aiming to coordinate research efforts and funding. In 2017, UK Research and Innovation published a cross-disciplinary mental health research agenda aimed at encouraging and strengthening research ‘that could fundamentally lead to: a better understanding of the broad determinants of mental health and mental illness across the life course, as well as a better understanding of mental health conditions and comorbidities; better knowledge of resilience or self-management of those conditions amongst individuals, communities or groups; improved diagnosis, care and treatment; improved experience of health and social care provision; more effective interventions and preventative methods; and better training of healthcare professionals’.^21^ This agenda highlighted priority areas for better understanding, including (a) mental health and mental health problems; (b) the connection between mental and physical health; (c) public health, prevention and well-being and (d) living with mental health problems. It included cross-cutting themes on effective interventions, technology and data, lifestyle and behaviour, inequalities, empowerment, ethics, confidentiality and trust. The agenda highlighted the importance of ‘social, cultural and community groups’ as an overall theme within research. However, it did not provide any specific details on priorities for researchers looking at SCCE. This is a pattern that can be traced across other mental health research agendas published by various funders and policy bodies, which generally allude to SCCE (in varying degrees) but provide little information on what aspects of this field of work should be prioritised.^22–25^ Although many of the broad research priorities within these agendas could by applied across the research on SCCE, this approach does not provide any specific nuance in terms of identifying questions that might be unique to SCCE. Further, these agendas provide a large number of questions with no prioritisation of which questions need addressing most urgently.

In response to the 2017 UK Research and Innovation mental health agenda, the project discussed here was funded as part of the cross-disciplinary mental health network programme, to develop and co-produce a new cross-disciplinary research agenda on SCCE and mental health. Funding was provided for a network that sought to bring together key stakeholders in SCCE and mental health, undertake activities to connect and support individuals and organisations to advance research, and seed fund promising research ideas. The funded network, MARCH, proposes that social, cultural and community assets build resilient communities and therefore lie at the heart of mental health. It aims to transform our understanding of how social, cultural and community assets affect mental health in the UK through supporting research into if and how these enhance public mental health and well-being, prevent mental illness and support those living with mental health conditions. This paper outlines the development and coproduction of a research agenda on SCCE and mental health by the MARCH Network.

## Method

### Developing the research agenda

This research agenda was ‘co-produced’ in that it gave equal weight to a broad range of stakeholders rather than privileging the opinions of researchers to provide a breadth of different perspectives, focus thinking onto research questions that could have an immediate and tangible impact on practice, avoid hierarchies or imbalances in power between different groups and provide a springboard for future collaborative projects. This co-production involved seeing all stakeholders as ‘leaders’ in the construction of the agenda rather than merely ‘consultees’; something that has been called for within mental health research agendas.^26^ Our approach follows similar processes and principles to other research agendas developed within health,^27,28^ including using an emergent and flexible design that offered initial open conversation and then gradually provided more process facilitation as we collated more information.

Phase 1 involved exploration. First, a core team of seven researchers mapped key stakeholders for this research area: researchers (including PhD students and early-career researchers); psychiatrists; other healthcare professionals and trainees; individuals with lived experience; lived experience groups; and members of the public, representatives from community and third-sector organisations, and individuals working within policy, commissioning or strategy. We then recruited 93 individuals within these different groups to join our research network as founding members and asked them to identify and recruit further members by using a snowballing approach. This led to a total recruitment of 1000 members within 6 months. We then actively engaged with these members following the underlying principles of agenda design by developing good social conditions between stakeholders, facilitating dialogue and encouraging respect for experiential knowledge. To do this, we provided communications material that introduced the work and perspectives of different groups, provided opportunities for online discussion forums through Twitter and online forums, and held face-to-face meetings and events for mixed groups with all stakeholders considered ‘experts’.

Phase 2 involved consultation. We prepared a simple anonymous online survey for stakeholders that asked individuals to propose research questions they felt need to be answered to advance practice in the field. We split responses into three sections: questions relating to the effects of SCCE, the content and components of assets or activities and the delivery and provision of assets and activities. This received 135 responses. We supplemented this survey with feedback from eight special interest groups that had organically developed among network members, and ideas put forward during two face-to-face events, each involving 90–100 participants.

Phase 3 involved integration and used a modified Delphi approach.^29^ The responses from phase 2 were grouped thematically into an initial draft agenda that was then put to the 93 founding members of the network. They responded to the draft with further suggestions and clarifications, and an iterative process followed until members were happy with the draft.

Phase 4 then involved returning the agenda to all network members and asking them to vote on which questions were of highest priority to each person's area of work. For this survey, we asked members to state which stakeholder group they represented. On completion, participants were given the option of re-answering the survey assuming a different role (e.g. first as a researcher and then as an individual with lived experience), and 15 participants opted to do so. In total, 284 responses were received in this phase, as shown in Table 2.

**Table 2:** Participants involved in the prioritisation phase of the research agenda development

Stakeholder group	*N*	%
Psychiatrists, other health professionals or trainees	45	15.7
Researcher (including PhD students and early-career researchers)	92	32.2
Individuals with lived experience, lived experience groups and members of the public	55	19.9
Representatives from community and third-sector organisations	59	20.6
Individuals working within policy, commissioning or strategy	33	11.5

This work received ethical approval from the University College London Research Ethics Committee (approval number: 14895/004), and all involved in the agenda process gave written or verbal informed consent.

## Results

### The research agenda

**Fig. 1:**
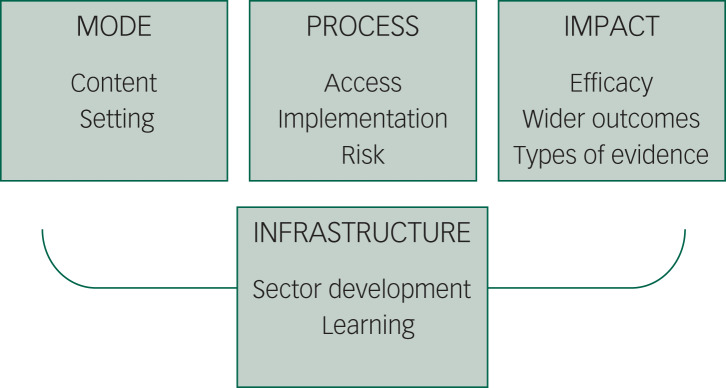
Summary of themes in the research agenda.

We identified four core themes within the agenda: the mode of engagement, the process of engagement, the impact of engagement and the infrastructure required to facilitate engagement (see Fig. 1). The full list of research questions in the agenda is shown in the supplementary material (Appendix 1) available at https://doi.org/10.1192/bjo.2020.133 and summarised below.

In relation to mode of engagement, two themes were identified. First, a number of research questions emerged relating to the *content* of community activities or engagement with community assets. These include questions on whether all types of SCCE are equally beneficial for mental health, if some modes of SCCE (e.g. live versus digital, or alone versus in groups) are more effective than others, whether certain types of facilitation or leadership of engagement are more effective than others, what amount of engagement is most beneficial for mental health, if quality of asset or activity content affects outcomes, what makes community activities and assets attractive for participants to engage and how activities and assets can be co-designed to meet the needs of individuals. There were also two main questions relating to the setting of SCCE: whether there is a difference in delivery and effectiveness depending on where engagement takes place, and exploring how ‘place’ shapes outcomes (for detailed research questions within these themes see Supplementary Appendix 1, sections 1–2).

In relation to the process of engagement, three themes were identified. A number of research questions emerged relating to access: how patterns of SCCE vary depending on individual characteristics, what the barriers or enablers to SCCE are among different groups and how we can create effective referral pathways to SCCE. There were also questions relating to implementation: whether activities and assets are being implemented appropriately, whether some people benefit more than others and what economic, political, social and cultural conditions are required for activities or assets to be a success. Finally, there were questions relating to risk*:* how we can minimise potential unintended consequences or adverse events arising from community activities, and how we can ensure safety and safeguarding of participants (for detailed research questions within these themes see Supplementary Appendix 1, sections 3–5).

In relation to impact of engagement, three themes were identified. Questions emerged relating to efficacy: the need for research exploring the effects of SCCE on prevention of mental illness and well-being among healthy individuals; research exploring the effects of SCCE on individuals with mild-to-moderate mental illness; research exploring the effects of SCCE for individuals with serious mental illness; and research exploring the effects of SCCE on the mental health of those delivering activities. There were also research questions on: wider outcomes, including under-researched outcomes related to mental health; secondary outcomes such as physical health, social determinants or behavioural risk factors; mechanisms by which SCCE affects mental health, comparing outcomes with those from non-SCCE interventions; and economic benefits. Finally, there were questions on the types of evidence being used: the use of under-utilised study designs such as ethnographies; analyses of population-level data and large-scale clinical trials; applying cross-disciplinary methods such as ecological monitoring, machine learning or geographical mapping; developing new theory to frame research on SCCE; using routinely collected data on health, education or behaviours; and comparing findings across different studies (e.g. through meta-analyses or developing standardised metrics or comparable measures for use across studies) (for detailed research questions within these themes see Supplementary Appendix 1, sections 6–8).

Finally, in relation to infrastructure required, two themes were identified. There were questions on sector development, including how we can train and support facilitators and practitioners, how we can support community organisations to work in this space, how we can expand the delivery of community activities and assets, how community activities can be made ‘sustainable’ for the future and how we could influence future policy developments to support this area of work. There were also questions on learning, including on how we can communicate better with the public, how we can share research better and how we can plan for new development opportunities with different sectors (for detailed research questions within these themes see Supplementary Appendix 1, sections 9–10).

### Prioritisation of research

We created ‘heat maps’ showing which specific questions within the themes outlined above received the highest numbers of votes from different stakeholder groups (see Supplementary Appendix 2). There were many points of agreement across all stakeholder groups, such as the importance of co-production within design, the need to undertake more efficacy research and the importance of how to make SCCE ‘sustainable’ for the future. Further, all groups gave relatively low priority to research into new types of evidence (with the exception of theory development among psychiatrists and other health professionals, researchers and policy makers), suggesting an agreement on the importance of prioritising research with immediate relevance to outcomes if we are to improve practice.

However, there were also noticeable differences (see Supplementary Appendix 2). Among psychiatrists and other health professionals, and community and third-sector organisations, there was particular interest in infrastructure, whereas among individuals working within policy, commissioning and strategy, there was particular interest in the effects of place on outcomes, the economic, political, social and cultural conditions required for activities or assets to be a success, and the economic benefits. Among individuals with lived experience and members of the public, there was also interest in how much engagement was most beneficial to mental health. In addition, this group along with researchers and representatives from community organisations highlighted the importance of research on how to train and support facilitators and practitioners.

## Discussion

This agenda confirms a number of the core themes that have emerged from previous high-level research agendas, including the UK Research and Innovation agenda, such as a focus on social and contextual factors, explorations of protective and resilience factors, improvements in public awareness and understanding, elucidation of mechanisms of action, considerations of place and participation, and barriers and enablers of engagement among different groups.^21^ However, it moves beyond these previous high-level agendas by seeing research on SCCE not just as a subset of a larger research agenda, but as an overarching topic that has its own specific research questions within it. Some of our priorities align with research recommendations from the National Institute for Health and Care Excellence on community engagement for improving health and well-being, including a focus on effectiveness (and cost-effectiveness, as prioritised by our policy stakeholders), a consideration of mechanisms and theory (as prioritised by our researchers and by psychiatrists and other health professionals) and a focus on collaborations and partnerships that can enable such work (as outlined in our sector development questions).^30^ The findings regarding barriers and enablers are also in line with recommendations in the updated Marmot Review, which recognises the importance of SCCE and community assets in addressing health inequalities,^31^ and the importance of SCCE in childhood is highlighted across a number of themes, echoing calls both from the Marmot Review and the World Health Organization's Social Determinants of Mental Health review.^31,32^ Further, our identification of questions relating to design, delivery and infrastructure is relevant given research suggesting that the majority of research in community-health sciences is skewed toward outcomes.^33^ These broader questions are therefore particularly highlighted as research gaps. Overall, our prioritisation exercise suggests there is a general consensus on the importance of key areas of research around efficacy, co-design and sustainability. However, it also highlights specific nuances among different stakeholders, suggesting the importance of considering the needs and requirements of different groups in developing research proposals or funding strategies. The agenda proposed is deliberately rich and diverse as different funders, research groups and stakeholders all have different areas of priority themselves. Therefore, different research questions in this agenda will speak to different groups. Nevertheless, the presentation of these diverse questions alongside one another in a unified research agenda should help future research to be more coordinated, and help to highlight the importance of considering different research questions (from the underlying research infrastructure to the tangible research impact) in parallel if we are truly to advance work in this field and the importance of further research into translation and innovation pathways.

Key strengths of the development of this research agenda include its open participatory, co-designed approach involving a large number of different stakeholders; the use of a range of modalities to elicit research questions from individuals; the use of a consensus approach to arrive at the final agenda; and the prioritisation of different research questions by different stakeholder groups. However, there are some limitations that affect the generalisability of the agenda presented. First, this work focused specifically on the UK context, so all stakeholders were from Great Britain or Northern Ireland. This enabled specificity when considering which research questions should be prioritised to inform practice, but it may mean that there are different or additional research questions that could or should be prioritised in other countries. Second, in prioritising research questions, stakeholders were asked to consider what would make the greatest difference to practice. Therefore, this prioritisation does not necessarily imply that those questions receiving lower votes are less important overall. Different funders may wish to use the agenda questions in new rounds of prioritisation to consider other priorities such as the advancement of research understanding, which may lead to higher prioritisation of those questions relating to topics such as types of evidence. Relatedly, this agenda specifically focuses on research, or how research can inform design and delivery. There may be other aspects to design and delivery and the broader environment that are priorities within practice or policy, but are not best explored through research, and therefore will not be captured within this agenda. In addition, the agenda focuses on where the key gaps are for future research, so areas that are already thought to be well covered are not shown. Finally, this agenda will inevitably evolve as new research is undertaken and as new challenges emerge. Therefore, it is intended to be a starting point for researchers and research funders rather than a constraint.

This agenda is timely as it considers the role of non-medical activities and assets within communities in supporting the prevention and management of mental illness. In the wake of the current COVID-19 pandemic, it is anticipated that there will be extreme pressure on mental health services in the coming months and years, leading to concerns that existing services will be unable to cope with demand. It is therefore of paramount importance that opportunities for providing mental health support from other sectors (including the cultural and community sectors) are properly explored to maximise the resources available. But it is also critical that the provision of such support remains evidence-based: that individuals are referred to appropriate, effective interventions and that the process of these referrals is transparent and suitable; and that there is sufficient infrastructure in place to support the organisations delivering social, cultural and community assets and activities. This research agenda identifies which research questions are going to be of highest priority in that effort. The agenda has now been taken forward within the research network to phases 5 and 6 of the agenda-developing process outlined by Abma and Broerse,^34^ which have involved developing a programme based on the research agenda and implementing the agenda through providing research funding targeted at the highest priority research questions presented. However, these phases should not be limited to the work of a single network, and so the agenda is presented here for researchers to continue implementing schemes that act on the findings. It is hoped that this will support the development of high-impact research within this valuable field of enquiry.

## Data Availability

The data that support the findings of this study are available from the corresponding author, D.F., upon reasonable request.

## Supplementary material

For supplementary material accompanying this paper visit http://dx.doi.org/10.1192/bjo.2020.133. click here to view supplementary material
