# Supplementary material for: Social, cultural and community engagement and mental health: cross-disciplinary, co-produced research agenda
Source: BJPsych Open. 2020 Dec 1;7(1):e3. doi: 10.1192/bjo.2020.133 (PMC7791563; doi:10.1192/bjo.2020.133)
Supplement: Supplementary file 1 [file S2056472420001337sup001.docx]

# Supplementary Materials

# Appendix 1: Full Research Agenda

## MODE OF ENGAGEMENT

- 1. CONTENT: Which aspects of community engagement are critical to mental health benefits?
  2. **Are all types of community engagement equally effective?**

Certain types of active community participation remain relatively under-researched, such as engagement with time banks and reading groups. Similarly, there remains a relatively small literature on receptive engagement with community assets such as visiting archives, going to a park, engaging with nature, attending cultural events, or visiting heritage sites, monuments or historic places. How does the magnitude of benefits from active participation compare with those from receptive engagement?

- 1. **Are some modes of community engagement more effective than others?**

Are there greater benefits from engaging alone or with others? How do results vary if engagement is with individuals with the same mental health conditions or with mixed participant groups, and are there added benefits from activities and assets being multi-generational or intersectional? How does in-person engagement compared to virtual or digital engagement affect results? Are there significantly stronger responses if activities and assets have been specifically tailored to the mental health needs of those engaging? Do individuals experience stronger responses from activities and assets if they feel they are contributing to rather than just receiving?

- 1. **Are certain types of facilitation of engagement more effective than others?**

Does having a leader vs engaging alone in activities and assets alter the impact? Does the role of expertise and experience (e.g. professional vs amateur vs volunteer) affect participants’ responses, and if so in what way? Are responses altered if a mental health professional or other professional is present at sessions? How important is the consistency of leadership for participants? How does this vary depending on the age of participants (e.g. for children vs adults) or their mental health needs? What is the importance of individual leadership in the scaling of activities or increasing engagement with assets? How are activities and their impact affected by whether or not leaders know specific details of the mental health needs of the group?

- 1. **What amount of engagement is most beneficial?**

What frequency of engagement leads to the greatest benefits for mental health? How do results vary depending on the amount of time spent engaging and the period of time over which that engagement occurs? How does this vary depending on the activity and the mental health outcomes being targeted? Are there particular benefits from individuals engaging in multiple types of activities or with multiple types of assets? Are there more or fewer benefits from frequent short episodes of engagement (e.g. multiple small exposures across a day) as from longer less frequent engagement (e.g. engagement for an hour or two once a week)?

**1.5 Does quality of content affect outcomes?**

How does the quality of leadership, teaching or facilitation affect responses? Does the quality of the process of engagement (e.g. rehearsing for a theatre production) matter more than the quality of the product of the engagement (e.g. the final theatre performance)? How do we measure quality?

- 1. **What makes community activities and assets attractive for participants to engage?**

What are the components of activities and assets that engage potential participants? Does having more agency or control or the chance to personalise the experience lead to better outcomes? Are there additional resources that enhance people’s experience of engaging? Is a final performance or showcase an appealing or off-putting component of an activity and how does this vary depending on the populations taking part? Does a formal peer support programme encourage people to engage? How much does enjoyment affect people’s decisions to continue engaging? What value do participants place on the individual attributes of an activity? What matters most to participants? Which attributes are they willing to trade for improvements in other attributes?

- 1. **How can activities and assets be co-designed to meet the needs of individuals?**

How can patient public involvement (PPI) and the involvement of individuals with lived experience of mental illness be used more effectively to inform the design of community activities and assets? How can activities continue to develop to stay relevant to participants?

1. SETTING: Are some settings more effective than others?
   1. **Is there a difference in delivery and effectiveness between settings?**

Do community activities work better in some settings than others, such as acute healthcare settings, other healthcare settings, individuals’ homes, schools, or the workplace? Do effects differ depending on whether engagement is online vs offline; indoors vs outdoors? Are participatory community activities (such as reading groups) more effective when delivered in community venues (such as libraries, heritage sites or museums)? How does setting vary in importance depending on the age of participants (e.g. children vs adults) and their mental health needs?

- 1. **How does place shape outcomes?**

How important is place in community activities for mental health? What is the nature of the relationship between individuals and their physical community environment around them? How important is place in building identity when engaging in activities or assets? How does the perception and rating of the physical space around them affect their engagement? Does engaging within nature enhance responses? Does engaging in activities or assets in asset-rich environments enhance responses? Do the aesthetics of place and aesthetic response to place affect mental health responses?

## PROCESS OF ENGAGEMENT

1. ACCESS: How can we enable community engagement?
   1. **How do patterns of community engagement vary?**

What proportion of people engage in different community activities or with different community assets? How does active participation and receptive engagement differ by individual demographic factors (such as age, gender and socio-economic status), cultural factors (e.g. ethnicity and cultural background), social factors (such as loneliness, isolation and social inequalities), existing mental health, and geographical factors? How do issues relating to intersectionality affect participation?

- 1. **What are the barrier/enablers to community engagement?**

How does community engagement depend on psychological capabilities (such as mental health, individual capital and awareness of activities or assets that are available), physical capabilities (such as disability or illness), social opportunities to engage (e.g. an individual’s social support, cultural understanding or perceived stigma, or a recommendation from a healthcare professional or family member/friend), physical opportunities to engage (e.g. the presence of activities or assets close by, the cost of engaging or the need to travel), reflective motivations (such as individual awareness of the evidence base) or automatic motivations (such as frequent public messaging). How are different people affected by different barriers/enablers?

- 1. **How can we create pathways to community engagement?**

Can we enhance general public engagement through developing public understanding or improving health communication on the benefits of community activities and assets? Can we develop strong referral pathways, not just relying on social prescribing but expanding to make the most of other opportunities? How might these referral pathways follow a patient journey, joining together primary, secondary and tertiary care? How would these referral pathways need to differ for different populations? Can we understand why some people take up referrals and others do not and when it is best to refer (i.e. what stage of mental health/where on the clinical pathway)? Can we manage transitions more effectively, such as supporting individuals when they move between settings (e.g. hospital to community)? How can we embed community engagement more effectively into individuals’ daily lives?

1. IMPLEMENTATION: How can community activities and assets be implemented effectively?
   1. **Are activities and assets being implemented appropriately?**

How suitable, acceptable and feasible are the activities and assets available or being developed? What is the reach and adoption of new activities or assets? Are activities able to maintain fidelity of delivery and receipt? Are activities quality assured (and what does this mean in practice)? Are activities that are being designed and implemented sustainable in the long-term? Are there sufficient assets within communities to support these activities? Why do some activities succeed whereas others close?

- 1. **What are the economic, political, social and cultural conditions required for activities or assets to be a success?**

How do factors such as austerity affect people’s engagement in their communities? What is the impact of major funding awards or cuts for community activities and assets on mental health? How are community activities and assets and their effect on mental health being affected by issues such as Brexit, immigration policies and increases in social inequalities? How can community organisations adapt to these factors?

- 1. **Do some people benefit more than others?**

Are the benefits of community engagement moderated by individual factors (such as sex/gender, genetics, age or stage in the life course, socio-economic status, training, social support, or past experience of community engagement)? Is there evidence of moderation by factors relating to mental health (e.g. current mental health, other ongoing treatment, or whether an individual is on a waiting list for treatment)? Is there evidence of moderation by geographical factors (such as neighbourhood characteristics, level of urbanisation, or the availability of activities or assets within the region)?

1. RISKS: What are the risks in delivering community activities for mental health?
   1. **Are there unintended consequences / potential adverse events from community activities?**

What are the risks of participants becoming dependent on activities or assets and how can this be managed? Are there risks of what happens to individuals once a programme ends? How should adverse events from community activities be flagged? Are there risks relating to individuals selecting community activities above other evidenced models of care and discontinuing or refusing alternative treatments? If a formal group involved in an activity continues to meet informally once an activity finishes, does this lead to risks of individuals no longer being properly supported?

- 1. **How can we ensure safety and safeguarding of participants?**

What is the best way to refer participants back to other mental health support once an activity finishes? Are there models of good practice for raising and reporting safeguarding issues? How can we ensure that individuals who are simultaneously engaging in community activities or assets and other mental health services receive joined-up care? How can we ensure support or signposting once an activity ends? Are there security risks for specific groups who may become the target or abuse or harm (e.g. activities targeted at ex-offenders or LGBT groups)?

## IMPACT OF ENGAGEMENT

1. EFFICACY: Which mental health populations can benefit from community engagement?
   1. **What are the effects of community engagement on primary prevention of mental illness?**

How could community engagement affect the risk of developing mental illness or support the enhancement of wellbeing amongst the general public? Are there particular benefits for engagement amongst specific groups such as children and adolescents, refugees and migrants, people from diverse ethnic and cultural backgrounds, individuals with learning difficulties, professional artists and arts practitioners, or professionals working in mental health?

- 1. **What are the effects of community engagement on mild-moderate mental illness?**

How does community engagement differentially affect individuals with different mental health conditions e.g. anxiety or depression? Do people with certain mental health conditions benefit more than others? Are there mental health benefits from community engagement amongst specific under-researched populations such as individuals at risk of a mental health crisis or with specific mental illness such as dissociative episodes or psychosomatic illness? Could engagement amongst individuals who have recovered from mental illness help them to remain well? What are the benefits for the mental health of individuals with physical illness?

- 1. **What are the effects of community engagement on severe mental illness?**

Are there benefits from community engagement for the management of symptoms in individuals with rare but serious mental illness, borderline personality disorder, schizophrenia, bipolar disorder, eating disorders, enduring mental illness, or treatment-resistant depression? Could community engagement help to reduce the risk of relapse or of developing comorbidities?

- 1. **What are the effects of community engagement on the mental health of those delivering activities or running assets?**

Does leading and organising community activities have adverse effects on the mental health of those delivering or leading activities? Are there particular adverse effects amongst individuals with their own history of mental illness? Do those delivering the activities experience any mental health benefits?

1. WIDER OUTCOMES: What are the wider effects of community engagement for mental health?
   1. **Which mental health outcomes are under-researched?**

At an individual level, how does community engagement affect under-researched aspects of positive psychological responses (such as resilience, nuanced aspects of individual wellbeing, or participant-selected goals) and under-researched aspects of mental illness (such as suicidality)? How does community engagement affect commonly used clinical outcome measures such as Clinician Rated Outcome Measures (CROMS), Patient Rated Outcome Measures (PROMS) and Patient Rated Experience Measures (PREMS)? At a community level, are there effects on social and community wellbeing, empowerment, and the mental health of wider networks such as family members and healthcare professionals if an individual engages more in their community?

- 1. **Which other related/secondary outcomes are under-researched?**

In addition to affecting mental health and wellbeing, does community engagement have wider benefits for factors such as psychological adjustment, physical health (such as related physical health outcomes or risk of or recovery from comorbid physical illness), social determinants of mental health (such as employability) or behavioural risk factors (such as risk-taking behaviours, teen pregnancy, violent behaviours, self-harm, or criminal offending)?

- 1. **What are the under-researched mechanisms by which community engagement affects mental health?**

How does community engagement affect under-researched cognitive mechanisms (such as individual beliefs, style of thinking and non-verbal communication), behaviours (such as levels of behavioural activation), physical responses (such as epigenetic responses or haptic perception and communication), and emotional responses (such as emotion regulation)?

- 1. **How do outcomes compare with outcomes from other interventions?**

How strong are the outcomes from community engagement compared with gold-standard psychological treatments (such as CBT or medication), medical interventions assessed by the National Institute for Health and Care Excellence, or other well-known health-promoting factors (such as diet or mindfulness and meditation)? What are the outcomes if individuals do not have access to community activities or assets?

- 1. **Are there economic benefits from community engagement?**

How cost-effective are community activities and assets for mental health? Are there wider economic benefits from community engagement for the health sector, such as in relation to health service utilisation, waiting lists and meeting current demand? Are there wider economic benefits for the voluntary and cultural sectors, or for society more generally (e.g. social services, housing, employment or economic growth)?

1. TYPES OF EVIDENCE: What types of evidence on the effects of community engagement are we lacking?
   1. **Which types of study design are under-utilised?**

Could our understanding be enhanced by more studies using currently under-utilised methodological approaches, such as studies focused on long-term outcomes of engagement, studies focused on ubiquitous daily engagement, larger-scale studies of effectiveness, or ethnographic studies of place-based implementation?

- 1. **How could we apply cross-disciplinary methods to answer research questions?**

What is the potential of using techniques such as natural experiments using cohort data, geographic information system mapping of assets, ecological monitoring, eye-tracking techniques or machine learning approaches to answer key research questions? How can we draw on work within the arts and humanities including creative research methods to address key research questions? How can we bring different methods together in truly interdisciplinary research?

- 1. **For which areas of research do we need to develop new theory?**

Which aspects of community engagement and mental health remain under-theorised? Can theory be applied and tested from other disciplines? Are there areas where new theory needs to be developed afresh?

- 1. **How could we use data more effectively?**

What opportunities are there for using electronic patient records, using routinely-collected data on utilisation of health or community services or using data from sectors such as local government services or education? Could technology such as smartphones to collect data on individual behaviours be used to collect useful data?

- 1. **How can we compare findings from different studies?**

Could we or should we develop standardised metrics or identify comparable measures for use across studies? Could we standardise evaluation processes for community organisations? Are we lacking certain meta-analyses, meta-syntheses, meta-ethnographies or other aggregations of data? Is there a value to applying taxonomies or schemata to categorise findings from different types of studies?

## INFRASTRUCTURE FOR ENGAGEMENT

1. SECTOR DEVELOPMENT: How can the community sector be supported to deliver this work?
   1. **How can we train and support facilitators/practitioners?**

What training would support practitioners in delivering activities or managing assets (such as formal courses or qualifications, or continuing professional development)? Could we and should we provide mental health support for community staff (e.g. individual training, continuing mental health support or peer support drawing on lived experience)? How can we ensure a workforce for the future (e.g. through identifying and recruiting new practitioners or the developing opportunities for and the capacity of volunteers)? How can we support individuals with lived experience to lead their own activities?

- 1. **How can we support organisations to work in this space?**

How can we build capacity within organisations (such as providing organisational training on using data, understanding evidence, or evaluation and research methods)? How can we develop more flexible funding models or strategies for organisations working in this space? Could we develop new models for employment to support the recruitment of staff to lead programmes? Can we use peer support or mentoring to support organisations, or build cross-sectoral learning (e.g. by building connections between similar organisations, sharing learning between different organisations, or sharing examples of good and bad practice)?

**9.3 How can we support the expansion of work?**

Are assets and organisations capable of working with more people if the demand increases? How can organisations be supported to deliver their work to more sites and reach more people? How can we help organisations to deliver programmes in areas where there are no or limited activities or assets at present? How can organisations be supported to branch out into new and ‘riskier’ types of delivery, whether new modes of delivery, working with new populations, or testing new programmes? How can we develop assets and access to assets within communities to support engagement?

- 1. **How can we ensure sustainable delivery of community activities and assets in the future?**

How can we support the continuation of community activities, such as through the continued provision or long-term funding of activities or the community assets they rely on? How can we connect with different sectors, such as work with the NHS or public health, or town-planning or social housing or development teams to ensure a place for community assets and activities in future developments? How can we ensure effective working between stakeholders in a way that builds sustainable relationships and avoids silos but whilst acknowledging sector boundaries? How can we plan for future developments and test new models of care to ensure organisations are adaptable? How can we ensure that potential harm to the community sector is identified and mitigated against so that assets and activities are not exploited?

- 1. **How can we work with and influence future policy developments to support this area of work?**

Which other sectors can we learn from in collaborating with policy makers to ensure the inclusion of this topic within new strategies and policies? What policy developments are needed to support the growth of this sector? How might ongoing changes within policy across different sectors (including welfare, housing, transport, communities, healthcare, social care, environment and the arts) affect the development of this work?

1. LEARNING: How can we support learning and communication between researchers and stakeholders?
   1. **How can we develop better communication with the public?**

How can we build public understanding and public health communication around the benefits of community engagement? What is the best language to be using and is the use of medical terms a help or a hindrance? How can we define and describe community engagement to include diverse activities and assets relevant to different groups of individuals?

- 1. **How can we share research better?**

How can we make research open and accessible and ensure that grey literature and evaluations are deposited in a way that means they can be shared? What is the best way to share research and evaluations amongst stakeholders? How can we gather evidence together in a way that supports its use? How can we avoid problems relating to pseudo-science or unsubstantiated claims about the benefits of community engagement?

- 1. **How can we plan for new development opportunities with different sectors?**

How can we connect research with policy and practice? How can we build new pathways of care across health, social care, education, third-sector and self-care? How can we stay ahead with developments in the design of healthcare and social care ? How can we build mental health into the development plans of creative industries? How can we engage more closely with other relevant sectors including education and local government?

# Appendix 2: Prioritisation according to different stakeholder groups

COLOUR KEY Percentage of stakeholders who rated the question a priority: 0-30%, 30-50%, 50-100%

|  | Psychiatrists, other health professionals or trainees | Researchers (including PhD students and ECRs) | Individuals with lived experience & members of the public | Representatives from community and third sector organisations | Individuals working within policy, commissioning or strategy |
| --- | --- | --- | --- | --- | --- |
| **MODE OF ENGAGEMENT** |  |  |  |  |  |
| **Content** |  |  |  |  |  |
| Research exploring if all types of community engagement are equally effective |  |  |  |  |  |
| Research exploring if some modes of community engagement are more effective than others |  |  |  |  |  |
| Research exploring whether certain types of facilitation/leadership of engagement are more effective than others |  |  |  |  |  |
| Research exploring what amount of engagement is most beneficial for mental health |  |  |  |  |  |
| Research exploring if quality of activity content affects outcomes |  |  |  |  |  |
| Research exploring what makes community activities and assets attractive for participants to engage |  |  |  |  |  |
| Research exploring how activities and assets can be co-designed to meet the needs of individuals |  |  |  |  |  |
| **Setting** |  |  |  |  |  |
| Research exploring if there is a difference in delivery and effectiveness depending on the setting of activities |  |  |  |  |  |
| Research exploring how ‘place’ shapes outcomes |  |  |  |  |  |
|  |  |  |  |  |  |
| **PROCESS OF ENGAGEMENT** |  |  |  |  |  |
| **Access** |  |  |  |  |  |
| Research exploring how patterns of community engagement vary depending on individual characteristics |  |  |  |  |  |
| Research into the barrier/enablers to community engagement amongst different groups |  |  |  |  |  |
| Research exploring how we can create effective referral pathways to community engagement |  |  |  |  |  |
| **Implementation** |  |  |  |  |  |
| Research exploring if activities and assets are being implemented appropriately |  |  |  |  |  |
| Research exploring if some people benefit more than others |  |  |  |  |  |
| Research identifying the economic, political, social and cultural conditions required for activities or assets to be a success |  |  |  |  |  |
| **Risk** |  |  |  |  |  |
| Research exploring how we can minimise potential unintended consequences / adverse events from community activities |  |  |  |  |  |
| Research exploring how we can ensure safety and safeguarding of participants? |  |  |  |  |  |
|  |  |  |  |  |  |
| **IMPACT OF ENGAGEMENT** |  |  |  |  |  |
| **Efficacy** |  |  |  |  |  |
| Research exploring the effects of community engagement on prevention of mental illness/wellbeing amongst healthy individuals |  |  |  |  |  |
| Research exploring the effects of community engagement on individuals with mild-moderate mental illness |  |  |  |  |  |
| Research exploring the effects of community engagement for individuals with serious mental illness |  |  |  |  |  |
| Research exploring the effects of community engagement on the mental health of those delivering activities |  |  |  |  |  |
| **Wider outcomes** |  |  |  |  |  |
| Research into under-researched outcomes related to mental health |  |  |  |  |  |
| Research into secondary outcomes such as physical health, social determinants or behavioural risk factors |  |  |  |  |  |
| Research into mechanisms by which community engagement affects mental health |  |  |  |  |  |
| Research comparing outcomes with those from other/’gold-standard’ interventions? |  |  |  |  |  |
| Research into economic benefits |  |  |  |  |  |
| **Types of evidence** |  |  |  |  |  |
| Research using under-utilised study designs such as ethnographies, analyses of population-level data and large-scale clinical trials |  |  |  |  |  |
| Research applying cross-disciplinary methods such as ecological monitoring, machine learning or geographical mapping |  |  |  |  |  |
| Research developing new theory to frame research on community engagement |  |  |  |  |  |
| Research using routinely-collected data on health, education or behaviours |  |  |  |  |  |
| Research comparing findings across different studies e.g. meta-analyses or developing standardised metrics or comparable measures for use across studies |  |  |  |  |  |
|  |  |  |  |  |  |
| **INFRASTRUCTURE FOR ENGAGEMENT** |  |  |  |  |  |
| **Sector development** |  |  |  |  |  |
| Research exploring how we can train and support facilitators/practitioners |  |  |  |  |  |
| Research exploring how we can support community organisations to work in this space |  |  |  |  |  |
| Research exploring how we can expand the delivery of community activities and assets |  |  |  |  |  |
| Research exploring how community activities can be made ‘sustainable’ for the future |  |  |  |  |  |
| Research focused on how we could influence future policy developments to support this area of work |  |  |  |  |  |
| **Learning** |  |  |  |  |  |
| Research exploring how we can communicate better with the public |  |  |  |  |  |
| Research exploring how we can share research better |  |  |  |  |  |
| Research exploring how we can plan for new development opportunities with different sectors |  |  |  |  |  |
